# The Implementation and Review of Cognitive Remediation Training for First Episode Psychosis in Singapore

**DOI:** 10.3389/fpsyt.2021.784935

**Published:** 2021-11-30

**Authors:** Nigel Ian Ming Chong, Yogeswary Maniam, Yi Chian Chua, Charmaine Tang

**Affiliations:** ^1^Yong Loo Lin School of Medicine, National University of Singapore, Singapore, Singapore; ^2^Early Psychosis Intervention Program, Institute of Mental Health, Singapore, Singapore

**Keywords:** schizophrenia, first-episode psychosis, early intervention programmes, cognitive remediation, cognition

## Abstract

**Objective:** Early intervention in patients with first episode psychosis (FEP) can improve cognitive abilities, with both short- and long-term benefits. In this paper, we describe the implementation and review of cognitive remediation training (CRT) in an Asian FEP population. The outcomes of the training are also evaluated and discussed.

**Methods:** This naturalistic paper describes in detail the real-life implementation and conduct of CRT in an early psychosis intervention service. One hundred and nine patients with FEP underwent a 24-session CRT programme, using Cogpack and Neuropsychological Educational Approach to Remediation. The program is evaluated with pre- and post-CRT assessment scores which included Montreal Cognitive Assessment and Brief Assessment of Cognition in Schizophrenia. The rates of improvement on these cognitive assessments were evaluated using paired *t*-tests, with statistical significance set at *p* ≤ 0.05.

**Results:** Of the 109 patients who underwent CRT, a total of 92 (84.4%) completed all 24 sessions. Paired *t*-tests between pre- and post-CRT assessments scores revealed that participants significantly improved on majority of the measures, including verbal memory, digit sequencing, and symbol coding.

**Conclusion:** As with other cognitive remediation programmes, CRT has shown to improve cognitive functioning in patients with FEP. The results support the use of CRT in an Asian context and may serve as guidance for the implementation of similar training programmes in other Asian early psychosis intervention services.

## Introduction

Schizophrenia and other psychotic disorders belong to a spectrum of primary psychotic disorders in the fifth edition of the Diagnostic and Statistical Manual of Mental Disorders (DSM-5) (1), and patients with psychosis present with neuropsychiatric disturbances including hallucinations, delusions, thought disorders, abnormal psychomotor behaviours, negative symptoms, cognitive impairments, and emotional disturbance (2). In the most recent Singapore Mental Health Study, Subramaniam and colleagues found that the lifetime prevalence of schizophrenia and other psychotic disorders in Singapore's adult population stands relatively low at 2.3% (3). However, schizophrenia is a chronic and severe mental disorder that has a profound impact on the individual and society. Although treatment has greatly advanced and outcomes may not be as uniformly negative as previously believed, over half of individuals with schizophrenia have intermittent but long-term psychiatric problems, and about 20% have chronic symptoms and disability (4). Following psychotic episodes, negative symptoms are commonly present and associated with cognitive impairments (5) and psychosocial disabilities. Therefore, it is not surprising to find unemployment at staggeringly high rates of 80–90% in some populations (6, 7). Furthermore, life expectancy is reduced by 10–20 years as compared to the general population (8, 9), largely due to increased cardiovascular-related morbidity and mortality (10), and contributed in part by an increased risk of suicide (11) and violence (12). These are the main reasons why this relatively infrequent disorder is responsible for the 6th largest share of disability adjusted life years (DALYs) in adults in Europe, and the 3rd largest of all brain disorders worldwide (13). In Europe, the total cost to society was 93.9 billion euros in 2010, and only the cost for mood disorders and dementia were higher amongst all brain disorders (13). In England, schizophrenia was found to cost society 11.8 billion pounds per year, equating to an average annual cost to society of about 60 thousand pounds per person (14).

Even on its first presentation, schizophrenia can lead to cognitive deficits similar to those found in chronic schizophrenia (15). Domains of cognition found to be affected include attention, working memory, verbal memory, and verbal fluency information processing (16). If left untreated, first episode psychosis (FEP) may result in poorer clinical and treatment outcomes (17), such as prolonged positive symptoms at 3, 6, and 12 months of treatment (18), lower rates of employment, and poorer global functioning (19).

While the severity of psychosis is concerning, appropriate interventions such as cognitive remediation (CR) can improve patients' cognitive abilities. Medalia and Choi described CR as an evidenced-based non-pharmacological intervention for cognitive deficits present in schizophrenia, encompassing cognitive drills or compensatory interventions to improve cognition (20). A randomised clinical trial conducted by Christensen studied the use of CR in conjunction with early intervention programmes, and showed that the addition of CR brought both short- and long-term improvements in both cognition and symptomology (21). With regard to symptomology, CR has been shown to exert greater effects in terms of negative symptoms (22, 23). CR has also shown improvement in functional outcomes (24), albeit with varying results (25).

To date, most of the publications on cognitive remediation training (CRT) in psychosis have been from Western countries, and literature from Asian societies have been limited. In a 2016 review by Tan and Liu (26) examining eight original papers on the research and clinical practice of CRT in China, the authors concluded that all papers reported some beneficial effects on cognitive functions, with three of them showing benefits on social functions. This study aimed to describe the conduct of CRT in an Asian early psychosis intervention service and evaluate the outcomes of CRT on individuals with FEP, by comparing pre- and post-CRT cognitive assessment scores.

## Method

The Singapore Early Psychosis Intervention Programme (EPIP) is a nationwide programme, which was launched in 2001 at the Institute of Mental Health (IMH), the only state psychiatric hospital in Singapore. Patients accepted into EPIP fulfil the following criteria: (a) age between 12 and 40 years old inclusive, (b) first-episode psychotic disorder with no prior or minimal treatment, and (c) psychotic disorder that is not secondary to a general medical condition or substance use. Psychotic Disorder is defined as meeting the Diagnostic and Statistical Manual of Mental Disorders IV-TR (DSM-IV-TR) (27) criteria for schizophrenia, schizophreniform disorder, schizoaffective disorder, delusional disorder, brief psychotic disorder, psychotic disorder not otherwise specified, or mood disorders with psychotic features. Intensive multidisciplinary care for patients presenting with first episode psychotic disorders is provided by EPIP in the initial 3 years, following which patients are transferred to other psychiatric services for continued care. Psychopharmacological treatment is based on a treatment algorithm that emphasises the use of antipsychotic monotherapy. Clozapine is considered in treatment-resistant patients who have failed at least 2 adequate trials of different antipsychotics, of which one should be a non-clozapine atypical antipsychotic. Depot medications are considered when medication non-adherence is identified. Adjunct medications such as antidepressants and mood stabilisers are used to treat comorbid anxiety and mood symptoms. Each patient has a case manager who provides supportive counselling, psychoeducation, and coordination of various services, while also ensuring continuity of care through the different phases of the illness. If required, patients are also referred for individual or group interventions with a psychologist, family therapist, occupational therapist (OT), or peer support specialist.

EPIP maintains an on-going database registered with the Singapore National Healthcare Group (NHG), capturing clinico-demographic data prospectively and maintaining data integrity through stringent and regular quality cheques. Ethics approval was obtained for the maintenance of this database. Patients are informed that routine data collected will be used for research and service evaluation purposes and those who disagree will still receive the relevant clinical services but not have their data collected. Each patient's duration of untreated psychosis (DUP) and diagnosis according to the DSM-IV-TR are assessed by their treating psychiatrist at baseline. Other rating scales are also administered at fixed time points (baseline, 3, 6, 12, 24, and 36 months). The Positive and Negative Syndrome Scale (PANSS) (28) is rated by the treating psychiatrist, and is a 30-item instrument consisting of three subscales—positive, negative, and general psychopathology. Each item is rated on a seven-point Likert scale, on which a higher score indicates a higher level of psychopathology. The Global Assessment of Functioning (GAF) scale (27) is rated by the treating psychiatrist, and consists of three subscales as well—total, symptom, and disability. Each subscale has a range of 0 to 100, on which the patient's level of functioning is rated, where higher scores indicate higher functioning.

CRT is offered as part of the programme to all suitable patients who have cognitive difficulties. CRT in EPIP makes use of the Cogpack (Marker Software^®^) (29) program and components from Neurocognitive Educational Approach to Remediation (NEAR), with the inclusion of group sessions to encourage social interactions and synthesise learning. Cogpack has shown to effectively improve cognitive functioning in several international randomised controlled trials (30–32), which contains structured neurocognitive exercises grouped into themes, such as attention, memory, speed of apprehension, visual motor, and reaction. These exercises are targeted at training certain domains of cognition that are typically hindered in schizophrenia (attention, working memory, verbal memory, verbal fluency, psychomotor speed, and executive function). NEAR takes on CR as a type of education (33), with stresses on short- and long-term goals that require patients to be insightful and motivated (34). There is also emphasis on patients to recognise the socio-emotional context of this approach and how it affects cognitive function (34). This can be done by organising group sessions that allow patients to interact with others and hone cognitive skills collectively (35). NEAR has shown to improve cognitive functions, namely processing speed (36), attention (37), and immediate learning and memory (38). The OTs running CRT have been trained in NEAR. As identified by Bowie et al. (39), Cogpack and NEAR are considered to be cognitive exercises, one out of the four core techniques in CR. Another core technique identified in the same study is the active involvement of a therapist, which would bring greater benefits for patients. This is practised in EPIP, with the help of experienced OTs, tailoring CRT sessions to each patient's progress and needs.

CRT in EPIP is an individualised 24-session programme that is delivered over a period of 3 months, with two sessions per week and each session lasting 1.5 h. It is delivered in a group with a maximum of five clients in a session. The same five clients are grouped together for all the 24 sessions. Through this, OTs were able to facilitate social skills norms and this helped to facilitate social communication, bonding and friendships amongst the group participants. A combination approach of both drill and practise as well as strategy coaching is adopted in the sessions. CRT initially starts out using Cogpack, making use of its repetitive drilling exercises. Cogpack is a software constituted by 64 exercises classifiable as domain-specific exercises and non-domain-specific exercises. The former act on individual skills such as verbal memory, verbal fluency, motor coordination, sustained attention, selective attention, working memory, and executive functions. The non-domain-specific exercises do not focus on one specific cognitive domain, but require the use and simultaneous involvement of aspects such as language, culture, and basic logical and mathematical skills. The exercises may be administered randomly and have their difficulty level adjusted by the computer on the basis of the performance in the course of sessions, so as to prevent the patient carrying out exercises that are too simple or excessively difficult (40). Exercises are selected by OTs to train individual domains of cognition, or combined exercises involving the use of multiple domains at once can be selected as well. OTs had gone through all the exercises in Cogpack and those that were not culturally relevant to the Singapore context were skipped but these were minimal. At times teaching of new words in relation to pictures were also facilitated by the OTs during the session as this promoted learning and facilitated recall which is linked to memory. OTs assess patients and tailor CRT in collaboration with the patient, by increasing or lowering the difficulty in accordance with each patient's progress. The exercises are built in a hierarchical manner starting with attention, processing speed, and reaction, and moving on to memory and problem solving. Cogpack displays the results after each exercise, showing patients their performance thus far. Encouragement and support are provided to patients. NEAR principles emphasise that the clients have a positive learning experience, they have a positive attitude about learning and that the games promote optimal cognitive functioning. Some of the NEAR games are Carmen Sandiego, Hot dog stand and Fripple Place. At EPIP commercial computer, board, or card Games using NEAR principles are brought in for patients who are assessed to have made sufficient progress. Bridging sessions are also a core component where patients discuss about the game and strategies they employ, and these are contextualised to their role and real-life situations. This not only facilitates social interaction amongst patients and the OT, but also transferability of skills learnt in sessions to real life situations (35). During and after each session, patients are required to record their own progress, by noting down what the activity involved and how they performed. They also set targets which serve as motivation for subsequent sessions. Individual review sessions are also conducted once a month with patients to highlight their progress, get feedback on how they are feeling and how they view the sessions and goals set for themselves.

Participants in the present study were receiving EPIP services and assessed to be suitable for CRT by their treating psychiatrist. Prior to commencement of CRT, level of participation in other psychosocial groups and source of motivation (whether the participation was of the patient's own volition, i.e., “self,” or if it was imposed upon by their caregiver, i.e., “enforced”) was also noted for each patient. There were no external rewards, monetary or otherwise, provided to incentivise patients to complete CRT. Pre- and post-CRT assessments, including the Montreal Cognitive Assessment (MoCA) (41) and Brief Assessment of Cognition in Schizophrenia (BACS) (42), were conducted by trained OTs and documented as well. In our naturalistic study, all consecutive patients who had presented to EPIP with FEP and underwent CRT, from February 2012–December 2019 inclusive, were included for analysis.

Statistical analyses were performed using IBM Statistical Package for Social Sciences (SPSS) 23. Continuous variables include age, DUP, PANSS positive, negative, and general psychopathology scores, and GAF symptom and disability scores. Categorical variables include gender, ethnicity, highest educational level, level of participation in other groups, source of motivation, baseline functioning status, and baseline DSM-IV diagnosis. Outcome measures include pre- and post-CRT assessments scores (including the MoCA and BACS). The rates of improvement on CRT assessments were evaluated using paired *t*-tests. Statistical significance was set at *p* ≤ 0.05.

## Results

The mean (SD) age for the overall study sample was 24.8 (5.6), of which nearly half (49.5%) were female and majority (77.1%) were Chinese. Out of the 109 patients who participated in CRT from 2012 to 2019, a total of 92 (84.4%) completed the full 24-session therapy. The baseline sociodemographic and clinical information of the sample, comparing between the groups who did and did not complete CRT, is presented in Table 1. Of note, the two groups differed significantly on their participation in other groups and source of motivation. Although the two groups also appeared to differ in terms of ethnicity, this is likely because for the Malay participants, all 13 of them had successfully completed CRT, as compared to the other ethnicities.

**Table 1:** Baseline sociodemographic and clinical characteristics of patients who participated in CRT.

	**CRT completed**		**Total (***n*** = 109)**	***P*** **-value**
	**Yes (***n*** = 92)**	**No (***n*** = 17)**		
Age – years, mean (SD)	24.8 (5.7)	24.7 (4.9)	24.8 (5.6)	0.916
**Gender – no. (%)**				
Female	44 (47.8)	10 (58.8)	54 (49.5)	0.405
Male	48 (52.2)	7 (41.2)	55 (50.5)	
**Ethnicity – no. (%)**				
Chinese	72 (78.3)	12 (70.6)	84 (77.1)	0.032*
Malay	13 (14.1)	0 (0.0)	13 (11.9)	
Indian	6 (6.5)	4 (23.5)	10 (9.2)	
Others	1 (1.1)	1 (5.9)	2 (1.8)	
**Highest education – no. (%)**				
Primary	1 (1.1)	1 (5.9)	2 (1.8)	0.390
Secondary	37 (40.2)	7 (41.2)	44 (40.4)	
Tertiary	54 (58.7)	9 (52.9)	63 (57.8)	
**Participation in other groups – no. (%)**				
Yes	53 (57.6)	4 (23.5)	57 (52.3)	0.010**
No	39 (42.4)	13 (76.5)	52 (47.7)	
**Motivation – no. (%)**				
Self	64 (69.6)	2 (11.8)	66 (60.6)	<0.001**
Enforced	28 (30.4)	15 (88.2)	43 (39.4)	
**Baseline functioning status – no. (%)**				
Age-appropriate role or employment	8 (8.7)	2 (11.8)	10 (9.2)	0.844
In training	1 (1.1)	0 (0.0)	1 (0.9)	
Unemployed	83 (90.2)	15 (88.2)	98 (89.9)	
**Baseline DSM-IV diagnosis – no. (%)**				
Schizophrenia spectrum	58 (64.4)	12 (70.6)	70 (65.4)	0.769
Mood disorders with psychotic features	6 (6.7)	0 (0.0)	6 (5.6)	
Delusional disorder	2 (2.2)	0 (0.0)	2 (1.9)	
Brief psychotic disorder and psychotic disorder not otherwise specified	17 (18.9)	4 (23.5)	21 (19.6)	
Others	7 (7.8)	1 (5.9)	8 (7.5)	
DUP – months, mean (SD)	9.3 (18.8)	4.7 (5.0)	8.5 (17.3)	0.324
**PANSS – mean (SD)**				
Total	82.2 (26.0)	85.9 (20.4)	82.8 (25.1)	0.578
Positive	20.5 (7.5)	22.2 (4.9)	20.8 (7.1)	0.380
Negative	18.9 (9.1)	18.8 (8.9)	18.9 (9.0)	0.967
General psychopathology	42.7 (14.0)	44.9 (12.2)	43.1 (13.7)	0.555
**GAF – mean (SD)**				
Total	40.9 (13.0)	39.9 (11.2)	40.8 (12.7)	0.774
Symptom	41.6 (13.4)	40.2 (10.6)	41.4 (13.0)	0.682
Disability	42.7 (12.1)	41.0 (11.1)	42.4 (11.9)	0.587

* *p ≤ 0.05*;

** *p ≤ 0.01*.

Normality was assumed due to the sample size, and paired *t*-tests (Table 2) between pre- and post-CRT assessments scores revealed that participants significantly improved on majority of the measures, except on the Semantic Fluency item. As the scores for the Trail Making Tests are operationalised by time taken to complete the task, the negative mean difference represents a positive result.

**Table 2:** Paired *t*-test results for CRT assessments scores.

	**Mean difference**	**95% CI**	***P*** **-value**
MoCA	2.02	1.41–2.63	<0.001*
Verbal memory	6.57	4.68–8.45	<0.001*
Digit sequencing	1.86	1.15–2.56	<0.001*
Token motor task	3.64	1.05–6.23	0.006*
Semantic fluency	1.28	−0.07 to 2.64	0.063
Symbol coding	3.95	2.18–5.71	<0.001*
Tower of london	1.65	0.84–2.47	<0.001*
BACS composite *z*-score	0.76	0.59–0.93	<0.001*
BACS composite *t*-score	7.60	5.92–9.28	<0.001*
Trail making test A	–4.88	–8.10 to –1.66	0.003*
Trail making test B	–15.94	–27.29 to –4.59	0.007*

* *p ≤ 0.01*.

## Discussion

Four core features of CR were identified in a white paper by Bowie and colleagues: a trained therapist, cognitive exercise trainings, attention to the development of strategies, and procedures to facilitate transfer to real world functioning (39). The current paper detailed the implementation of CRT in EPIP and illustrated how these four core components were fulfilled. The CRT sessions were held twice a week with a total training duration of 36 h, meeting the effective intensity and durations found by Bowie et al. (39), whereby effective programmes call for two to three sessions per week, with total training durations ranging from 20 to over 40 h. Our duration surpassed recommendations by Hooker et al. (43) that 25–30 h of cognitive training is required for cognitive benefits. A mixed approach of drill and practise with strategy coaching was utilised. The bridging sessions facilitated transferability of skills taught in the classroom-like setting to the real world. This contextualisation increased patients' interest, enabling them to make connections and linkages to their everyday functioning, and aided them in forward planning and goal setting.

A major strength of our study was that we naturalistically examined a large cohort of patients with FEP who underwent CRT, looking at data collected over a span of seven years. We were able to utilise data from the EPIP database, which is an on-going registry capturing information prospectively. The variables collected are clearly defined and objectified using standard psychiatric rating instruments; and data integrity is subjected to stringent and regular quality cheques by the nation's relevant governing body. However, as with naturalistic studies, the OTs running the sessions were the ones who conducted the pre and post assessments. As such, there could have been potential for bias, even though this was minimised by ensuring that well-established, standardised and validated test instruments were used, and the OTs conducting the cognitive assessments had the necessary prerequisite training.

Our results were consistent with existing literature which indicates that CRT has beneficial effects on the cognitive abilities of patients with early psychosis. A limitation to our study was that there was no follow up data to see if the cognitive changes were sustained after completion of CRT. A study conducted by Buonocore et al. (44) demonstrated stable cognitive abilities 5 years after treatment completion, with the exception of psychomotor speed and coordination. Buonocore made use of Cogpack, similar to our CRT, which provides some assurance of its long-term results. It was interesting to note that participants who completed CRT had significant improvements on all measures except for semantic fluency. For semantic fluency, participants are required to generate as many words as possible from a given semantic category within a limited time. The role of executive functioning increases with the degree of retrieval difficulty, and more strategic planning is required. Impairments in semantic fluency are typically either related to blunt executive functioning (lack of sustained retrieval management), or to a breakdown of semantic knowledge (associated with semantic/conceptual memory disruption or/and storage shrinkage) (45). Our findings appeared to differ from other studies where improvements in semantic fluency were noted post-CRT (46). It is possible that the MoCA was not sensitive enough to pick up changes in an Asian FEP population where albeit being English-literate, English may not be the main conversational language. It is certainly worthwhile exploring this further in a controlled trial with a larger sample size.

There was a high completion rate of CRT, and amongst those who completed CRT, a significant majority were self-motivated and participated in other psychosocial group activities. Although this may indicate an intrinsic motivation which naturally leads to better outcomes, it is noteworthy that motivation was the only significant factor associated with completion of CRT, and suggests that services should place emphasis on increasing both intrinsic and extrinsic motivation during the course of CRT, and examine this relationship further with more robust methodologies. In a meta-analysis by Medalia and Saperstein (47), outcomes of NEAR have strong correlations to participants' motivation. As NEAR is part of CRT, EPIP has adopted various methods to raise the motivation of participants. Patients can voice their opinions and wishes, and are actively involved in goal-setting and decision-making for each session. Having autonomy and being empowered to share their thoughts and feelings have shown to raise participants' intrinsic motivation (48), as well as promote interest (49), thereby keeping them engaged with CRT. Each session is also facilitated by an actively involved OT, who adjusts sessions according to each patient's individualised progress, and utilises an appropriate scaffolding approach as the patients advance through the sessions and cognitive tasks (49). The sessions are contextualised and personalised for each patient, engaging and allowing patients to have a sense of control over their learning process. The level of difficulty is adjusted accordingly for “just enough” challenge to be presented to the patients for every session and this helps to promote self-perceived competence which increases their motivation in the sessions.

A systemic review by Reser et al. (50) had indicated that CRT improved working memory in younger but not older participants. It was also mentioned that reasoning, problem solving, and working memory were strongly predictive of within-domain improvement, and that training task progress was a strong cross-domain predictor of cognitive outcome. A meta-analysis on computer-assisted CR (CACR) by Grynszpan et al. (51) noted significant effect sizes on processing speed, attention, working memory, and verbal learning and memory. A more recent multi-outcome meta-analysis by Kambeitz-Ilankovic et al. (52) studied outcomes of CACR alone and CACR with supplementary human guidance, which demonstrated similar outcomes to Grynszpan, with the addition of social cognition, reasoning, and global cognition. Looking to an Asian population, an evaluation on CACR was conducted in Hong Kong and found that CACR improves neurocognition (53).

What aids the efficacy of CRT, which patient characteristics would ideally benefit from it, and its long-term effects is still being explored and debated upon (54). While our study has demonstrated how CRT can be implemented in an Asian early intervention service and adds to the limited body of literature assessing CRT in an Asian FEP population, further studies will need to be conducted to maximise the gains from CRT.

## Data Availability Statement

The raw data supporting the conclusions of this article will be made available by the authors, without undue reservation.

## Author Contributions

NC: writing–original draft. YM: conceptualisation, methodology, investigation, writing–review & editing, and supervision. YC: formal analysis, data curation, and writing–review & editing. CT: conceptualisation, methodology, writing–review & editing, and supervision. All authors contributed to the article and approved the submitted version.

## Conflict of Interest

The authors declare that the research was conducted in the absence of any commercial or financial relationships that could be construed as a potential conflict of interest.

## Publisher's Note

All claims expressed in this article are solely those of the authors and do not necessarily represent those of their affiliated organizations, or those of the publisher, the editors and the reviewers. Any product that may be evaluated in this article, or claim that may be made by its manufacturer, is not guaranteed or endorsed by the publisher.
